# Type 2 diabetes prevalence varies by socio-economic status within and between migrant groups: analysis and implications for Australia

**DOI:** 10.1186/1471-2458-13-252

**Published:** 2013-03-21

**Authors:** Marian Abouzeid, Benjamin Philpot, Edward D Janus, Michael J Coates, James A Dunbar

**Affiliations:** 1 Greater Green Triangle University Department of Rural Health, Flinders University and Deakin University, P.O. Box 423, Warrnambool, Victoria 3280, Australia; 2 Department of Epidemiology and Preventive Medicine, Monash University, Melbourne, Australia; 3 Department of Medicine, Northwest Academic Centre, The University of Melbourne, Western Hospital, Melbourne, Australia

**Keywords:** Type 2 diabetes, Migrant, Socio-economic status

## Abstract

**Background:**

Ethnic diversity is increasing through migration in many developed countries. Evidence indicates that type 2 diabetes mellitus (T2DM) prevalence varies by ethnicity and socio-economic status (SES), and that in many settings, migrants experience a disproportionate burden of disease compared with locally-born groups. Given Australia’s multicultural demography, we sought to identify groups at high risk of T2DM in Victoria, Australia.

**Methods:**

Using population data from the Australian National Census and diabetes data from the National Diabetes Services Scheme, prevalence of T2DM among immigrant groups in Victoria in January 2010 was investigated, and prevalence odds versus Australian-born residents estimated. Distribution of T2DM by SES was also examined.

**Results:**

Prevalence of diagnosed T2DM in Victoria was 4.1% (*n* = 98671) in men and 3.5% (*n* = 87608) in women. Of those with T2DM, over 1 in 5 born in Oceania and in Southern and Central Asia were aged under 50 years. For both men and women, odds of T2DM were higher for all migrant groups than the Australian-born reference population, including, after adjusting for age and SES, 6.3 and 7.2 times higher for men and women born in the Pacific Islands, respectively, and 5.2 and 5.0 times higher for men and women born in Southern and Central Asia, respectively. Effects of SES varied by region of birth.

**Conclusions:**

Large socio-cultural differences exist in the distribution of T2DM. Across all socio-economic strata, all migrant groups have higher prevalence of T2DM than the Australian-born population. With increasing migration, this health gap potentially has implications for health service planning and delivery, policy and preventive efforts in Australia.

## Background

The aetiology of type 2 diabetes mellitus (T2DM) involves complex interactions between genetic, developmental, evolutionary and environmental influences [1]. Current theories suggest that the developing foetus controls its physiology to be most adaptive to the predicted postnatal environment. For example, intrauterine deprivation is thought to result in irreversible epigenetic and developmental plasticity processes that facilitate enhanced fat storage and reduced metabolism. This confers a survival advantage if postnatal conditions are poor. Such programming would, however, confer increased risk of metabolic disease on exposure to environments of excess, as may occur in societies changing their diet, and with exposure to obesogenic environments after migration to developed countries [1]. Globalisation has resulted in growing high-risk migrant populations in many developed nations. Differential vulnerability to and prevalence of T2DM among some migrant groups has important implications for prevention policy and clinical practice worldwide.

In some settings, diabetes prevalence among some migrant groups differs from that of locally-born populations [2-4]. Absence of differences [5] and variable associations depending on the ethnicity of the locally-born groups [6] have also been described. In multicultural Australia, several databases contain relevant information, but few analyses of diabetes distribution among migrant groups have been published [7-16]. These are now either dated, consider only a few countries/regions of birth, have insufficient power to detect immigrant differences, apply national prevalence estimates by broad migrant groupings and prevalence rates in countries of origin to local population birthplace data, and / or do not age-adjust. As migrants comprise differing proportions of the population of each Australian state and territory, and regions of origin and time of migration varies [17,18], national trends may not necessarily reflect T2DM epidemiology at a state level. Diabetes prevalence among migrants from a given region may differ from that in the country of origin [3,5,19,20], but not universally [19,21].

Some socio-economic indices vary between migrant groups [17]. In Australia, prevalence of diabetes and its modifiable risk factors are known to vary with some facets of socio-economic status (SES) [10,12,15,22,23]. We are not aware of any Australian studies that have investigated SES differences in T2DM prevalence within or between migrant groups.

Having over a quarter of its residents born overseas [18], the Australian state of Victoria is culturally diverse, making it an appropriate setting for such a case-study. We investigated variations in the prevalence of diagnosed T2DM in migrant and socio-economic groups in Victoria, using population demographic data from the 2006 national census of the Australian Bureau of Statistics (ABS) and diabetes data for those with diagnosed T2DM registered with the National Diabetes Services Scheme (NDSS).

## Methods

The NDSS is funded by the Australian Government and administered through Diabetes Australia. It provides access to subsidised diabetes-related products such as blood glucose meter strips. Registration forms include basic demographic questions and clinical details such as diabetes type (validated by either a medical practitioner or diabetes educator, and mandatory for registration) and self-reported date of diabetes diagnosis. Approximately 80-90% of Australians with diagnosed diabetes mellitus are registered with NDSS [24]; membership is free.

De-identified data were obtained for Victorian residents registered with NDSS on 20^th^ January 2010. Datafields included country of birth, Aboriginal and Torres Strait Islander (ATSI) status, age group, sex, type of diabetes (this paper considers only T2DM), and residential postcode at time of data extraction. The 0.5% of registrants with missing postcodes (*n* = 890) were excluded.

### Data classification and manipulation

As statistical power necessitated analysis at regional rather than country level, countries were categorised into regions using the ABS’s *Standard Australian Classification of Countries*[25]. Based on geographic proximity, the broad-level classification system comprises nine regions. These vary in the size and number of constituent nations, ranging from eight countries comprising North-East Asia to 53 in the Americas. The classification system includes Australia in the group Oceania and Antarctica. In order to examine Australian-born people separately, we recategorised this group into ‘Australia’ and ‘Oceania’ (the latter also including Antarctica). The Australian-born group includes Australian-born Indigenous people. Given socio-cultural and developmental similarities between Australia and New Zealand and differences between New Zealand and other Oceanic countries, we generated two additional region-of-birth variables post hoc, denoted ‘New Zealand’ and ‘Pacific Islands,’ in which New Zealand was a distinct category separate from the Pacific Islands in Oceania. Registrants with missing country of birth were classified as unknown.

Age (categorised as 0–29 years, 30–39, 40–49, 50–59, 60–69, 70–79, 80+) denotes age of registrants at time of data extraction. As current diabetes prevention programs in Victoria target non-Indigenous high-risk individuals aged 50 years and over [26], we also generated a dichotomous age variable (> = 50 years, <50 years). Age at diagnosis was not used as this information was unavailable for 39% of registrants with T2DM, whereas age at time of data extraction was complete.

Residential area socioeconomic deprivation was obtained from the ABS *Socio-Economic Indexes for Areas*[27], using the *Index of Relative Socio-Economic Disadvantage* (IRSD) postal area scores. A composite measure of area-based socio-economic disadvantage, the IRSD incorporates 17 census items including proportion of people with low incomes, low education and unemployed. ABS assigns IRSD scores to collection districts, standardised against a mean of 1000 and with a standard deviation of 100. These collection districts are then combined to yield postal area scores that approximate to postcodes used in this study. Low scores represent relatively high levels of disadvantage and vice versa. Postal areas were additionally ranked into SES quintiles, representing IRSD scores 765–960, 961–993, 994–1025, 1026–1058, and 1059–1142.

### Generation of a data set representing the entire Victorian population by type 2 diabetes status

Data for people with diagnosed T2DM were obtained directly from the NDSS database. These totals were then subtracted from ABS census data to obtain the estimated numbers without T2DM. The data fields were region of birth, age group, sex, T2DM status (i.e. has or does not have T2DM) and postcode. IRSD scores and quintiles were assigned based on postcode. Region rather than country of birth was used due to the statistical power requirements described above and to the fact that ABS census data include random errors to preserve confidentiality – this requires that the number of groups retrieved from the census data is minimised to reduce the error inherent in the total Victorian dataset. Because of data collection time differences, a few census groups were smaller than their NDSS counterparts (mostly with region of birth unknown), requiring a negligible increase in the census denominator.

### Statistical analyses

**Figure 1:**
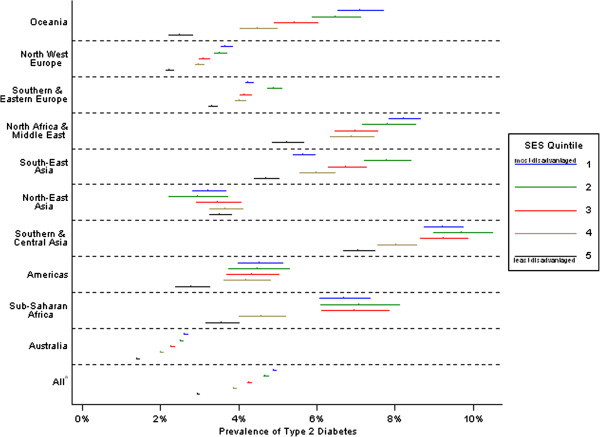
**Age-adjusted prevalence rates of type 2 diabetes with 95% CI bars by region of birth and SES, males.**^a ^includes region of birth unknown.

**Figure 2:**
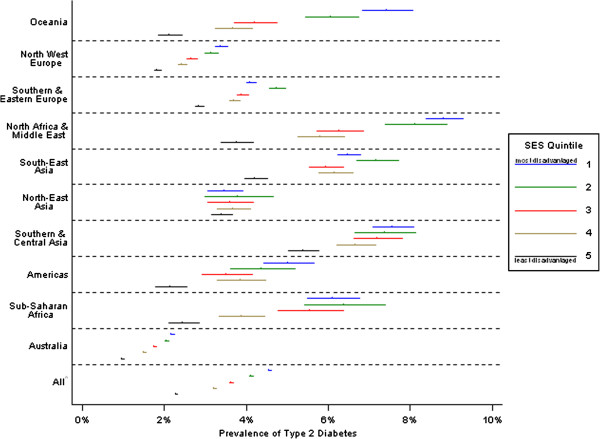
**Age-adjusted prevalence rates of type 2 diabetes with 95% CI bars by region of birth and SES, females.**^a ^includes region of birth unknown.

Statistical analyses were performed using Stata 12.0. Chi-square and two-sided independent *t*-tests were used to compare Victorian NDSS registrants with T2DM with known and unknown region of birth. Registrant characteristics were profiled using descriptive statistics (Table 1), and regions of birth were compared using chi-square tests and linear regression. Logistic regression was used to calculate odds ratios of diagnosed T2DM for each region versus the Australian-born group (Tables 2 and 3), both crudely and adjusted for age group and additionally SES quintile in separate models. Adjusted prevalence rates were derived from a model that included age group as a co-variate. Using models adjusted for IRSD quintile, age-group and sex, tests of interaction sought to identify any variation in the association of T2DM with region of birth by these variables. *Contrasts,* a technique of reducing the number of pairwise comparisons to control type 1 errors, were used to test the significance of T2DM prevalence based upon the category of IRSD quintile. Sex-specific prevalence rates, adjusted for age-group, were estimated for SES quintiles within regions (Figures 1 and 2). ATSI data are not presented due to very small numbers and limited statistical power.

**Table 1:** Demographic characteristics of NDSS registrants with Type 2 diabetes mellitus, Victoria 2010

	**Males**			**Females**		
**Region of birth**		**Age < 50**	**SES**^**a,c**^		**Age < 50**	**SES**^**a,d**^
	**n (%)**	**% (95% CI)**^**b**^	**Mean (95% CI)**^**b**^	**n (%)**	**% (95% CI)**^**b**^	**Mean (95% CI)**^**b**^
**Oceania**	1473 (1.5)	22.0 (20.0-24.2)	991 (988–995)	1253 (1.4)	27.9 (25.4-30.4)	987 (983–990)
**North-West Europe**	8824 (8.9)	4.6 (4.2-5.1)	1006 (1005–1008)	7243 (8.3)	5.0 (4.5-5.5)	1002 (1000–1003)
**Southern & Eastern Europe**	14599 (14.8)	2.5 (2.3-2.8)	991 (990–992)	12423 (14.2)	2.4 (2.1-2.6)	988 (987–990)
**North Africa & Middle East**	2899 (2.9)	13.1 (12.0-14.4)	977 (974–980)	2318 (2.6)	17.8 (16.3-19.4)	966 (962–969)
**South-East Asia**	3272 (3.3)	15.4 (14.2-16.7)	984 (982–987)	3943 (4.5)	17.6 (16.4-18.8)	979 (976–981)
**North-East Asia**	1100 (1.1)	14.2 (12.2-16.4)	1032 (1028–1036)	1229 (1.4)	19.4 (17.3-21.7)	1031 (1028–1035)
**Southern & Central Asia**	3423 (3.5)	25.8 (24.4-27.3)	1005 (1003–1008)	2554 (2.9)	22.5 (20.9-24.2)	1004 (1001–1006)
**Americas**	714 (0.7)	13.2 (10.9-15.9)	995 (990–1001)	651 (0.7)	14.1 (11.7-17.0)	987 (982–993)
**Sub-Saharan Africa**	1043 (1.1)	19.2 (16.9-21.7)	999 (994–1003)	856 (1.0)	19.6 (17.1-22.4)	992 (987–996)
**Australia**	29218 (29.6)	12.8 (12.5-13.2)	1001 (1000–1001)	24857 (28.4)	13.8 (13.4-14.2)	997 (996–998)
**Unknown**	32106 (32.5)	7.9 (7.6-8.2)	999 (998–1000)	30281 (34.0)	13.1 (12.7-13.5)	995 (994–996)
**TOTAL**	98671 (100)	9.7 (9.6-9.9)	998 (998–999)	87608 (100)	12.1 (11.9-12.3)	994 (994–995)
**Overseas**	37347 (37.9)	8.9 (8.6-9.2)	996 (995–996)	32470 (37.1)	9.8 (9.5-10.1)	991 (991–992)
**Pacific Islands**	603 (0.6)	22.9 (19.7-26.4)	974 (967–980)	611 (0.7)	30.8 (27.2-34.5)	975 (969–981)
**New Zealand**	870 (0.9)	21.4 (18.8-24.2)	1003 (999–1007)	642 (0.7)	25.1 (21.9-28.6)	998 (993–1003)

^a ^measured using the Index of Relative Socio-economic Disadvantage score; ^b ^95% confidence interval; ^c ^excludes 522 males for whom residential area SES scores were not available; ^d ^excludes 368 females for whom residential area SES scores were not available.

**Table 2:** Crude and adjusted prevalence rates (%) and prevalence odds ratios (OR) by region of birth, males

**Region of birth**	**Victorian population size**^**a**^	**Observed**	**Age-adjusted**^**b**^	**Observed**	**Age-adjusted**^**b**^	**Age- and SES-adjusted**^**c**^
	**n**	**%**	**%**	**OR (95% CI)**^**d**^	**OR (95% CI)**^**d**^	**OR (95% CI)**^**d**^
**Oceania**	39802	3.7	4.9	2.2 (2.1-2.3)	2.5 (2.4-2.7)	2.6 (2.5-2.8)
**North-West Europe**	141754	6.2	3.0	3.8 (3.7-3.9)	1. 5 (1.4-1.5)	1.5 (1.4-1.5)
**Southern & Eastern Europe**	142711	10.2	4.1	6.4 (6.3-6.6)	2.0 (2.0-2.1)	2.0 (2.0-2.1)
**North Africa & Middle East**	37540	7.7	7.1	4.7 (4.6-4.9)	4.0 (3.8-4.2)	4.0 (3.8-4.2)
**South-East Asia**	74102	4.4	5.9	2.6 (2.5-2.7)	3.2 (3.0-3.3)	3.1 (3.0-3.3)
**North-East Asia**	41127	2. 7	3.5	1.6 (1.5-1.7)	1.7 (1.6-1.8)	1.9 (1.8-2.0)
**Southern & Central Asia**	54555	6.3	8.4	3.8 (3.7-3.9)	5.0 (4.8-5.2)	5.2 (5.0-5.4)
**Americas**	19252	3.7	4.0	2.2 (2.0-2.4)	2.0 (1.8-2.2)	2.0 (1.9-2.2)
**Sub-Saharan Africa**	22072	4.7	5.3	2.8 (2.6-3.0)	2.8 (2.6-3.0)	2.9 (2.7-3.1)
**Australia**	1682160	1.7	2.1	Referent	Referent	Referent
**TOTAL**^**e**^	2421553	4.1	4.1	n/a	n/a	n/a
**Overseas**	572915	6.5	4.2	3.9 (3.9-4.0)	2.1 (2.1-2.2)	2.2 (2.1-2.2)
**Pacific Islands**	7829	7.7	10.1	4.7 (4.3-5.1)	6.5 (5.9-7.1)	6.3 (5.7-6.9)
**New Zealand**	31973	2.7	3.6	1.6 (1.5-1.7)	1.8 (1.7-1.9)	1.9 (1.7-2.0)

^a ^based on 2006 Victorian census counts, ^b ^adjusted for age group; ^c ^adjusted for age group and SES quintile (n = 4997 missing data); ^d ^95% confidence interval; ^e ^includes region of birth unknown.

**Table 3:** Crude and adjusted prevalence rates (%) and prevalence odds ratios (OR) by region of birth, females

**Region of birth**	**Victorian population size**^**a**^	**Observed**	**Age-adjusted**^**b**^	**Observed**	**Age-adjusted**^**b**^	**Age- and SES-adjusted**^**c**^
	**n**	**%**	**%**	**OR (95% CI)**^**d**^	**OR (95% CI)**^**d**^	**OR (95% CI)**^**d**^
**Oceania**	41062	3.1	4.4	2.2 (2.1-2.3)	2.9 (2.7-3.1)	3.0 (2.8-3.2)
**North-West Europe**	143311	5.1	2.6	3.7 (3.6-3.8)	1.6 (1.6-1.7)	1.7 (1.6-1.7)
**Southern & Eastern Europe**	146632	8.4	3.8	6.4 (6.3-6.6)	2.5 (2.4-2.5)	2.4 (2.4-2.5)
**North Africa & Middle East**	35047	6.6	6.7	4.9 (4.7-5.1)	4.8 (4.6-5.1)	4.7 (4.5-5.0)
**South-East Asia**	91986	4.3	5.9	3.1 (3.0-3.2)	4.1 (4.0-4.3)	4.0 (3.9-4.2)
**North-East Asia**	50406	2.4	3.5	1.7 (1.6-1.8)	2.3 (2.1-2.4)	2.6 (2.4-2.7)
**Southern & Central Asia**	44767	5.7	6.7	4.2 (4.0-4.4)	4.8 (4.6-5.0)	5.0 (4.8-5.2)
**Americas**	20967	3.1	3.7	2.2 (2.1-2.4)	2.4 (2.2-2.6)	2.4 (2.2-2.6)
**Sub-Saharan Africa**	22895	3.7	4.4	2.7 (2.5-2.9)	3.0 (2.8-3.2)	3.1 (2.9-3.3)
**Australia**	1754144	1.4	1.7	Referent	Referent	Referent
**TOTAL**^**e**^	2513054	3.5	3.5	n/a	n/a	n/a
**Overseas**	597073	5.4	3.8	4.0 (3.9-4.1)	2.5 (2.5-2.5)	2.5 (2.5-2.6)
**Pacific Islands**	9027	6.8	9.5	5.1 (4.6-5.5)	7.6 (6.9-8.3)	7.2 (6.6-7.9)
**New Zealand**	32035	2.0	2.9	1.4 (1.3-1.5)	1.8 (1.7-2.0)	1.9 (1.8-2.1)

^a ^based on 2006 Victorian census counts; ^b ^adjusted for age group; ^c ^adjusted for age group and SES quintile (n = 4072 missing data); ^d ^95% confidence interval; ^e ^includes region of birth unknown.

Because region of birth was not known for 33% (*n* = 62,387) of NDSS registrants with T2DM and 7% of Victorian residents (*n* = 328,315), we examined the generalisability of our results using multiple imputation to estimate missing region of birth. Ten datasets were imputed, for men and women separately, using chained equations for region of birth (multinomial logistic regression) and IRSD quintile (ordered logistic regression), adjusting for each other as well as age group and T2DM status. These tests gave similar results to our analyses that ignored missing data, giving confidence that there is no selection bias inherent in excluding those with missing region of birth.

Flinders and Monash Universities granted ethics approvals (IDs 4713 and 2009001942, respectively). NDSS staff extracted data and granted permission for its analyses*.*

## Results

### Demographic characteristics of Victorian residents with type 2 diabetes mellitus

There were 186,279 Victorian residents with diagnosed T2DM registered with NDSS, of whom 53.0% were male. Age, sex and mean area IRSD scores were similar between those for whom region of birth was known and unknown (data not shown).

Of T2DM registrants with known birthplace, the largest proportion was Australian-born (Table 1). Slightly more females (12.1%) than males (9.7%) were aged under 50 years (χ^2^_[1]_ = 263, *p* < 0.01), with pronounced regional variation.

Mean crude IRSD score differed by birthplace (Table 1). After adjusting for age group (data not shown), regional differences in mean crude IRSD score were observed for both males (*p* < 0.01) and females (*p* < 0.01). When examined as pair-wise comparisons, there were highly significant (*p* < 0.01) differences in IRSD scores between Australian-born and all overseas-born groups, except for Sub-Saharan Africa (males and females), New Zealand (males and females) and the Americas (males only).

### Prevalence of type 2 diabetes in Victoria

Crude T2DM prevalence in Victoria was 4.1% among males and 3.5% among females, with much variation by region of birth (Tables 2 and 3). All overseas-born groups were significantly more likely to have diabetes than the Australian-born referent, crudely and after adjusting for age and IRSD score (Tables 2 and 3). While odds ratios changed markedly after age adjustment, additionally adjusting for IRSD score had a smaller effect. Among both males and females, fully adjusted odds ratios were greatest for those born in the Pacific Islands, Southern and Central Asia and North Africa and the Middle East.

As statistically significant interactions between gender and region of birth were found between Australian-born and most migrant groups, separate analyses were performed for males and females. Tests of interaction also demonstrated significant interactions between SES quintiles and region of birth for all groups except Sub-Saharan Africa and New Zealand.

Within each region, age-adjusted T2DM prevalence rates differed by SES (Figures 1 and 2). Prevalence rates were relatively stable across levels of SES for North-East Asia, but within all other groups, those living in the most disadvantaged areas had the highest prevalence rates. For both males and females, strong social gradients were particularly evident within Oceania, North-West Europe and Australia. SES differences were also apparent between migrant groups and the Australian-born population. Migrant groups generally had higher rates of T2DM than Australian-born individuals of the same SES at all levels of SES. Additionally, for many migrant groups, the least deprived still had higher prevalence than Australian-born men and women living in the most deprived areas.

## Discussion

Our results demonstrate large differences in prevalence rates of T2DM within and between migrant groups in Victoria, and a large migrant health gap compared with the Australian-born population. Differences between groups persist even after allowing for effects of socio-economic disadvantage. The proportion with diagnosed T2DM aged under 50 years varies markedly by region of birth.

Over 20% of registrants born in Oceania and in Southern and Central Asia were aged under 50 years when NDSS data were extracted. Disease onset and diagnosis may have occurred much earlier when these subjects were even younger. At diagnosis in Australia, 22.5% of people with T2DM were aged less than 45 years [23]. Our results suggest that this proportion may vary by region of origin. We support recommendations for earlier commencement of diabetes screening, at age 35, for Pacific Islanders, Indians and Chinese [28].

Even after adjusting for age and SES, prevalence odds of T2DM were higher in all migrant groups compared with the Australian-born group. The reasons underlying this cannot be elucidated from our data. International literature suggests that effects and mediators of the nature/nurture interaction may be contextual – for example, prevalence of diabetes among male Tunisian migrants to France was lower than rates in Tunisia but similar to that of French-born men in France. The apparently protective effect of migration among this group was partly mediated by factors such as physical activity and smoking [5]. Studies of Japanese immigrants and their offspring in the United States have, however, demonstrated the complex interplay between lifestyle factors promoting visceral adiposity and insulin resistance, and unmasking impaired beta-cell function in genetically susceptible individuals [20]. Second generation Japanese Americans had higher prevalence of diabetes than both Japanese in Tokyo and the rest of the USA adult population [20]. It is possible that similar factors are at play for some migrant groups in Victoria. Future research should seek to elucidate any such influences in the local context.

In this study prevalence differed by region of birth and was further influenced by SES level (Figures 1 and 2). In many developing and transitional countries, diabetes prevalence increases with SES whereas the reverse is true in developed nations. For some migrant groups in our study, these diabetes–SES associations are not as clear but it is not possible to infer causation. There are multiple factors at work. Circumstances underpinning relocation may influence migrants’ demographic characteristics (e.g. skilled workers vs. refugees). Age at and time of migration may determine extent of acculturation and attendant lifestyle and behavioural risk exposures, choice of residential area, and socio-economic position. Migrants exhibit much mobility in the first decade after relocation to Australia, and large differences exist between migrants on some socio-economic indicators, such as employment status, based on time of migration [17]. Additionally, numerous factors other than SES may influence where people live. Migrant groups may cluster in particular residential areas, for reasons unrelated to SES.

### Comparison with other studies

Our findings for Southern and Eastern European born people agree with higher reported diabetes prevalence among Greeks and Italians compared with Australian-born people [14]. Our higher age-adjusted T2DM prevalence rates for all migrant groups, however, contrasts with recent reports from the Victorian Health Monitor [15] showing no differences. Some of our results also differ from earlier Australian analyses including reports of higher diabetes prevalence rates among some but not all migrant groups in the 2000 AusDiab study [8] and recent analyses of the New South Wales [13] and National Health Surveys [7,12,16]. Comparison between studies is difficult as immigrant categories and age ranges differed and there are some differences in inclusion criteria for diabetes while we used clinician-validated data for NDSS registrants with diagnosed T2DM. Rising prevalence of obesity [12], and changing migratory patterns altering Australia’s demographic landscape [18] since studies such as AusDiab were conducted, also hinder comparisons.

Our findings confirm a strong area-based social gradient in T2DM prevalence overall [10,22,23], but differ from another recent Victorian study [15]. SES was based on that of the residential area at the time of data extraction and not current individual circumstance, which has been reported to be associated with diabetes prevalence in Victoria [15]. Notably, social factors other than those considered in our study may influence disease risk. SES is a multifaceted construct and its various aspects, such as educational attainment, assets and access to healthcare, may well exert differential effects.

### Strengths and limitations

In the NDSS, clinician validated diagnosis renders misclassification of presence or type of diabetes less likely than patient self-report. NDSS is a contemporary database, continuously updated with new registrants. Interstate migrations are identified by monitoring the State in which NDSS access occurred. Deceased registrants are removed through annual linkages with the National Death Index or following notification by relatives (pers. comm.). NDSS is considered among the best available national data sources for estimating overall prevalence of diagnosed diabetes [24], but recent migrants may be under-represented as only Australian or New Zealand citizens and Australian permanent residents qualify for government Medicare Cards, an NDSS eligibility requirement. Temporary NDSS registration may be granted to visitors from countries with reciprocal healthcare agreements with Australia. Additionally, those unaware of the scheme or managed by diet alone may be under-represented [24].

There are further reasons for possible underestimation of prevalence. NDSS only captures diagnosed cases registered with the scheme. Recent Victorian data indicate that for every three people with diagnosed diabetes, there is one undiagnosed [15]. On the other hand the Victorian population denominator was based on 2006 census data, and may result in overstated prevalence rates. Given the proportion with missing birthplace data, our migrant-specific prevalence rates represent minimum estimates and may further underestimate the true prevalence of diagnosed T2DM in Victoria. It is possible that rate of diagnosis may differ by region of birth, possibly influenced by factors such as health-seeking behaviours and contact with the local healthcare system, and that among those with diagnosed diabetes, the rate of registration with NDSS may also vary.

Our Australian-born reference population includes ATSIs, a subgroup known to have a higher burden [10,29] and earlier age at diagnosis of T2DM [29]. Analysing them separately was not possible due to uncertainty surrounding the size and demographic characteristics of the Victorian Indigenous population. Census counts indicate that only 0.6% of the total Victorian population identify as Indigenous; including ATSIs in our referent group is therefore unlikely to markedly influence our findings.

This study captures only first-generation immigrants, and it is not possible to infer ethnicity, ancestral background or any other ethnic parameter that may influence diabetes risk. The Australian-born group contains second and later generation Australians, who may retain behavioural and genetic risk profiles of the ancestral ethnicity. An example of that comes from a study of Indians in Singapore reporting higher diabetes prevalence among those born in Singapore to Indian-born parents than in the Indian-born immigrants [30].

Heterogeneity of regional classifications may also mask differences within groups as some regions comprise culturally, linguistically, religiously and developmentally diverse nations. In Australia, self-assigned ethnicity is not widely collected. As it was not available in the NDSS data set, analysing or interpreting our data in the context of population racial or ethnic composition within each migrant group is not possible.

## Conclusions

Our findings have implications for policy and preventive efforts in Australia. Identifying population sub-groups at greater risk of T2DM enables mapping of their size and geographic distribution, guiding health service planning. Such information also has clinical implications by facilitating identification and screening of those from known high-risk groups, and aiding planning and development of culturally appropriate health education, which may improve some outcomes among those with established diabetes [31].

The International Diabetes Federation recommends diabetes prevention using both the population approach and targeting those at high risk [32]. Our findings suggest that preventive efforts must specifically target lower socio-economic groups, in addition to high-risk migrant groups at all levels of SES. Future research should seek to untangle how the risk associated with being a migrant is linked to socio-economic circumstance in the local context, and to investigate behavioural risk factor profiles of migrants from varying socio-economic strata within each region of origin group.

Awareness of migrant groups at high risk of T2DM needs to be further increased among health professionals. Screening for diabetes should start at younger ages for some immigrant groups. Migrant communities need to be aware of their risks and services available to them. While diabetes has featured as a National Health Priority Area in Australia for some time, closing the migrant health gap that exists for this disease must now come to the forefront of clinical, public health and political agendas.

## Abbreviations

ABS: Australian Bureau of Statistics; AOR: Adjusted odds ratio; ATSI: Aboriginal and Torres Strait Islander; CI: Confidence interval; IRSD: Index of Relative Socio-economic Disadvantage; NDSS: National Diabetes Services Scheme; OR: Odds ratio; SES: Socio-economic status; T2DM: Type 2 diabetes mellitus.

## Competing interests

The authors declare that they have no competing interests.

## Authors’ contribution

MA designed the study, assisted with data analysis, wrote manuscript; BP designed the study, analysed data, edited manuscript; EJ designed the study, edited manuscript; MC designed the study, prepared data, assisted with data analysis, edited manuscript; JD conceived, designed and supervised the study, edited manuscript. All authors read and approved the final manuscript.

## Pre-publication history

The pre-publication history for this paper can be accessed here:

http://www.biomedcentral.com/1471-2458/13/252/prepub
